# Diagnostic Utility of the Neutrophil-Lymphocyte Ratio and Absolute Lymphocyte Count in Distinguishing Thyroiditis vs Other Benign Causes of Thyroid Enlargement

**DOI:** 10.7759/cureus.81379

**Published:** 2025-03-28

**Authors:** Gaurav Sharma, Ashutosh Kumar, Ankita Mittal, Richa Singh, Nikhil Chaudhary

**Affiliations:** 1 Department of Pathology, Teerthanker Mahaveer Medical College and Research Centre, Moradabad, IND

**Keywords:** fine needle aspiration cytology (fnac), goitre, inflammation, thyroid cytopathology, hashimoto’s thyroiditis

## Abstract

Introduction: Thyroid disorders range from localized lesions, such as colloid goiter, to autoimmune thyroiditis and can present as a tumour mass. Despite a huge number of lesions, they should be classified into two basic categories: one with a diffuse pattern of involvement and the other that results in thyroid gland nodules. Thyroid nodules are frequently found in clinical settings, accounting for 4-7% of the adult population. Thyroiditis, along with hyperplasia, generally affects the entire gland, which is linked to diffuse expansion of the thyroid. Many disorders, including thyroid conditions, can be effectively managed, and their progression can be largely prevented through a well-functioning immune system. Neutrophil-lymphocyte ratio (NLR) and absolute lymphocyte count (ALC) are markers of systemic inflammatory response. There is a paucity of literature available on the correlation of NLR and ALC with thyroiditis compared to other benign thyroid disorders. Current research was conducted to depict the diagnostic utility of NLR and ALC in differentiating between thyroiditis and other benign causes of thyroid enlargement.

Methodology: This is a case-control study conducted from November 2022 to April 2024 (for a period of 18 months). Data were collected from patients with thyroid swelling who underwent fine needle aspiration cytology (ultrasonography-guided or non-USG-guided) from the cytopathology section. Data on the hematological parameters of the same patients were collected from the hematology section after considering the inclusion and exclusion criteria. Data were entered into an MS Excel sheet (Microsoft® Corp., Redmond, WA) and evaluated using SPSS (version 24; IBM SPSS Statistics for Windows, Armonk, NY).

Results: In the current research, a total of 136 samples were taken. Out of 136 samples, 121 (89.0%) were women, and 15 (11.0%) were men. Hashimoto’s thyroiditis (HT) constituted 18.4% (25 cases), and the rest were benign causes of thyroid enlargement, as a control group (81.6%, 111 controls). The mean age for HT was 36.44±12.81 years, while it was 38.45±12.62 years in the control group. The mean total leukocyte count (TLC) for HT was 8651.20±2378.03 cells/cumm, and for the control group, it was 7367.30±2204.37 cells/cumm. The p-value for TLC was 0.011. The mean neutrophil and lymphocyte percentages for HT were 65.44±11.92 and 28±11.31, respectively, while in the control group, it was 68.23±10.44 and 25.91±8.77, respectively. The results obtained for the NLR were 3.17±2.84 in HT and 3.26±2.38 in the control group. The mean of ALC in HT was 2379.32±1137.53 cells/cumm, and in the control group, it was 1843.14±674.35 cells/cumm. The p-value for ALC was 0.002. The results were statistically significant for the total leukocyte count and ALC.

Conclusion: The gold standard in the assessment of thyroid disorders is histopathological examination, as the NLR is not specific for thyroid disorders, and this should not be considered as a diagnostic test alone. The NLR, although a cheap and noninvasive marker, has limited value in the diagnosis of thyroid disorders, while the ALC provides additional specificity to this inflammatory profile, which showed significant results in current research. More studies and other inflammatory markers with large sample sizes are needed to establish the correlation between ALC and different thyroid disorders.

## Introduction

Thyroid nodules are generally observed in 4-7% of the population when examined by palpation and observed in 50% of the population when assessed by ultrasonography [1]. Multinodular goiter (MNG) presents as thyroid enlargement with nodules. MNG is considered a benign disease with a low risk of malignancy [2]. Few researchers have advocated that the risk of malignancy associated with MNG is comparable to that of solitary thyroid nodules; nevertheless, fine-needle aspiration (FNA) of MNG is restricted because it presents with multiple nodules [3].

Lymphocytic thyroiditis is attributed to the autoimmune pathologic mechanism. Papillary thyroid cancer has been commonly observed to be associated with lymphocytic thyroiditis [4]. Histopathologic and radiological assessment are essential in distinguishing between these thyroid diseases [5]. Immunity plays a crucial role in controlling the progress of many disorders, inclusive of carcinoma [6]. A straightforward approach to immune response includes general hematological investigation markers in the form of total leucocyte count and differential leucocyte count, which are considered of prognostic and diagnostic value in numerous carcinomas [7].

To the best of our knowledge, there is a paucity of published scientific literature exploring the association between the neutrophil-lymphocyte ratio (NLR) and absolute lymphocyte count (ALC) in differentiating between various thyroid disorders. Current research was conducted to evaluate the association between NLR and ALC in thyroiditis versus other benign thyroid conditions.

## Materials and methods

A case-control study was conducted from November 2022 to April 2024 for a duration of 18 months. Ethical clearance was obtained from the institutional ethical clearance committee with reference number TMU/IEC/2021-22/55. Data were collected from the cytopathology and hematology section of the central diagnostic research laboratory. For the inclusion criteria, data were collected from adult patients with thyroid enlargement in the cytopathology lab and on fine needle aspiration cytology (FNAC) reported as Bethesda category II. Patients not willing to participate in the study and patients refusing to undergo the investigation of complete blood count (CBC) required for NLR and ALC were excluded. Data included the total leucocyte count (TLC), differential count, and ALC from CBC. The NLR and ALC were calculated from the differential count. The NLR was calculated by dividing the neutrophil percentage (neutrophil %) count by the lymphocyte percentage (lymphocyte %) count. Informed consent was obtained from the patients for both the FNA procedure and the CBC blood sample collection. The current research was carried out on 136 thyroid patients. FNA samples from these patients were collected. CBC samples were then collected from those patients who were diagnosed cytopathologically as thyroid Bethesda category II. After obtaining the FNA samples, glass slides were made and stained with May-Grünwald Giemsa (MGG) and hematoxylin and eosin (H&E) stains. For CBC, 2 mL of blood was collected in an ethylenediaminetetraacetic acid vial and run in a Beckman Coulter hematology analyzer (DxH 900) to obtain different CBC parameters. Comparison of thyroiditis and other benign causes of thyroid enlargement as a control group was made based on TLC, differential count, NLR, and ALC. In the current research, we identified two categories: Hashimoto’s thyroiditis (HT) and the control group (which included other benign causes of thyroid enlargement such as colloid goitre, nodular hyperplasia with solitary nodule, and benign thyroid lesions). Various parameters, such as gender, age, TLC, neutrophil%, lymphocyte%, NLR, and ALC, were evaluated. Data were collected and entered into an Excel spreadsheet (Microsoft® Corp., Redmond, WA). Data were evaluated using Statistical Product and Service Solutions (SPSS, version 24; IBM SPSS Statistics for Windows, Armonk, NY). Continuous variables were demonstrated by mean and standard deviation. Categorical variables were depicted by frequency and percentage. Statistical analysis was performed using a chi-squared test and one-way ANOVA. The level of significance was considered as 0.05.

## Results

In the current research, the calculated sample size was 136, of which 121 (89.0%) were women and 15 (11.0%) were men. HT constituted 18.4% (25 cases) of the 136 cases, and the rest were other benign causes of thyroid enlargement as the control group (81.6%, 111 cases). In overall thyroid disorders, a female preponderance was noted. As shown in Table 1, the mean age for HT was 36.44±12.81 years. It was higher (38.45±12.62 years) in the control group. The mean TLC for HT was higher (8651.20±2378.03cells/cumm) than in the control group (7367.30±2204.37 cells/cumm). The increased TLC in HT can be attributed to the inflammatory response caused by thyroid disease. The results were statistically significant for TLC, with a p value of 0.011. The mean neutrophil % and lymphocyte % for HT were 65.44±11.92 and 28±11.31, respectively, while, in the control group, the mean neutrophil % was 68.23±10.44, and the mean lymphocyte % was 25.91±8.77. The NLR was 3.17±2.84 in HT and 3.26±2.38 in the control group. The mean ALC was higher in HT (2379.32±1137.53 cells/cumm) than in the control group (1843.14±674.35 cells/cumm), perhaps due to an increase in chronic inflammatory states associated with HT. The results were statistically significant only for TLC and ALC, with p values of 0.011 and 0.002, respectively.

**Table 1:** Comparison between various parameters (age, TLC, N%, L%, NLR, ALC) with various benign thyroid disorders and thyroiditis. ALC: absolute lymphocyte count; NLR: neutrophil-lymphocyte ratio; N%: neutrophil percentage; L%: lymphocyte percentage; TLC: total leukocyte count TLC and ALC were statistically significantly higher in HT as compared to other benign thyroid conditions, whereas other parameters (age, N%, L%, and NLR) were not statistically significant. Statistical analysis was performed using the chi-square test and one-way ANOVA. The level of significance was considered as 0.05.

Parameters	Hashimoto’s Thyroiditis Mean ± SD	Control Group (Other Benign Causes of Thyroid Enlargement) Mean ± SD	P value
Age (years)	36.44 ± 12.81	38.45 ± 12.62	0.473
TLC (cells/cumm)	8651.20 ± 2378.03	7367.30 ± 2204.37	0.011
N%	65.44 ± 11.92	68.23 ± 10.44	0.241
L%	28 ± 11.31	25.91 ± 8.77	0.293
NLR	3.17 ± 2.84	3.26 ± 2.38	0.869
ALC (cells/cumm)	2379.32 ± 1137.53	1843.14 ± 674.35	0.002

## Discussion

The association between cancer and its surrounding inflammatory microenvironment has been well-documented in the literature. Researchers have highlighted how tumors trigger inflammatory reactions, which influence disease progression and prognosis [8]. Among the various inflammatory markers, the NLR has emerged as a significant predictor of the overall survival in carcinomas [9]. Elevated NLR, coupled with decreased lymphocyte counts and neutrophilic leukocytosis, intensifies inflammatory responses in the tumor milieu across multiple cancer types [10].

Given its superficial position, the thyroid gland is accessible for direct physical assessment, FNA, and surgical biopsy [11]. Enlargement of the thyroid gland is a common clinical complaint across all ages, whether inflammatory or neoplastic. NLR and platelet-lymphocyte ratio (PLR) are now widely used criteria in studies of various disorders, including acute and chronic inflammatory pathologies. The impact of NLR, PLR prognosis, survival, and illness indices of various inflammatory disorders and cancers has been evaluated. Several investigations showed profound associations [12].

In the current research, the mean age was 36.44±12.81 for HT. Aksu et al. conducted a similar study, including 81 cases along with 54 healthy individuals. They observed a mean age of 32.9±12.3, which was lower as compared to the current research because their study mostly consisted of healthy controls [13]. Resber et al. observed a mean age of around 47.68±15.93 for the patient group (HT), which was higher than in the current research (36.44±12.81 for HT), with a p-value of 0.473, which was statistically insignificant (i.e., there is no significant age difference between those with HT and those with other benign causes of thyroid enlargement). In comparison, thyroid diseases are more common in younger age groups, which explains the lower mean age in the HT in our study, as it included controls with benign thyroid disorders, while other studies primarily focused on healthy controls [14]. 

In the current research, the mean TLC for HT was higher (8,651.20 cells/cumm) as compared to our control group (7,367.30 cells/cumm), with a p-value of 0.011, which was of substantial statistical significance. The higher TLC can be attributable to the induced inflammatory responses in thyroid disorders. Aksu et al. demonstrated a mean TLC in the patient group of 7.89±1.42 (109/L), which was slightly lower compared to the present research. The reason for this may be that their research contained 81 HT cases, whereas the current research only had 25 cases. Additionally, the controls in their study were healthy cases, whereas our study assessed benign thyroid disorder cases [13].

In the present research, the mean neutrophil % of HT was 65.44, and for the control group, it was noted around 68.23, with a p-value of 0.241, which was not of statistical significance in neutrophil % among both groups. Matei et al. observed a mean neutrophil % in the control group (benign thyroid nodule) of around 62.17±11.29, which was slightly higher (68.23±10.44) as compared to the current research, with an insignificant p-value of 0.241. This is because they took a larger sample size and compared malignant and benign thyroid nodules in their study [15].

In contrast, the mean lymphocyte % of HT was 28.08, and for the control group, it was 25.91, with a p-value of 0.293. This indicates no statistically significant difference in lymphocyte % between the groups. Matei et al. observed a mean lymphocyte % in the control group of 28.20±8.41, with a p-value of 0.027. This was not comparable to our control group, which was slightly lower (25.91), with a p-value of 0.293, which was not substantially significant [15].

The mean NLR of HT observed in the current research was 3.17, and the mean for the control group was 3.26, with a p-value of 0.869, indicating no statistically significant difference in NLR between both groups. Aksu et al. reported that the mean NLR in the patient group was 2.37±1.46, with a statistically significant p-value of 0.003. This is because they undertook healthy controls, and their sample size was 81 cases of HT [13]. Matei et al. reported a mean NLR in the control group of 2.49±1.94, which was not comparable to our control group mean of 3.26. The ALC of HT was noted as 2379.32 cells/cumm, whereas the mean for the control group was 1,843.14 cells/cumm, with a significant p-value of 0.002, indicating a substantially significant difference in ALC among both groups [15]. Aksu et al. noted a mean ALC in the patient group of 2.24±0.63 (109/L). In the current research, this value was nearly the same and statistically significant, but it was statistically not significant in their study [13]. Resber et al. reported a mean ALC of 2.4 (103/μL) in euthyroid HT. Our study yielded similar results with significant findings. The substantial significance observed may be attributed to their comparison of euthyroid HT with healthy individuals and a larger sample size in their study [14]. Matei et al. reported a mean ALC in the control group of 2.08±0.69 (103/μL). This value was less in our research control group because we compared HT inflammatory disease with benign thyroid lesions, whereas they studied malignant and benign thyroid nodules [15].

## Conclusions

Our study highlights the diagnostic utility of the TLC and ALC in distinguishing thyroiditis from other benign causes of thyroid enlargement. Elevated NLR values are indicative of the other inflammatory processes in the thyroid; however, our study failed to demonstrate a significant role in differentiating HT from other benign causes of thyroid enlargement. Other studies, however, have demonstrated the utility of this parameter. This difference may be due to a difference in the study designs because we included thyroid enlargement due to other benign pathologies as the control, whereas other studies used healthy volunteers as the control, and ALC provides additional specificity to this inflammatory profile. These hematological parameters offer a noninvasive, cost-effective, and readily available means of differential diagnosis. However, although TLC and ALC are useful adjuncts, they should be integrated with clinical evaluation and other diagnostic modalities for optimal accuracy. Further research and larger cohort studies are required to validate such results and to refine the thresholds for clinical application, ensuring reliable and precise differentiation in diverse patient populations.
